# Supplementary material for: Neuronal TORC1 modulates longevity via AMPK and cell nonautonomous regulation of mitochondrial dynamics in C. elegans
Source: eLife. 2019 Aug 14;8:e49158. doi: 10.7554/eLife.49158 (PMC6713509; doi:10.7554/eLife.49158)
Supplement: Source code 1. [file elife-49158-code1.rtf]

A.) .IJM code for MitoMAPR-1.0var SampleIdARRAY = newArray();var ObjectsARRAY = newArray();var JunctionsArray = newArray();var JunctionsPerNetworkArray = newArray();var NetworksARRAY = newArray();var ObjectLengthARRAY = newArray()var mitoAreaARRAY = newArray();var MitoPartAreaArray = newArray();var CoverageArray = newArray();var CellAreaArray = newArray();open();imgName=getTitle();run("8-bit");  waitForUser ("make a selection");  run("Clear Outside");  selectWindow(imgName);         rename("ROI.tif");         selectWindow("ROI.tif");         run("Save");         // MitoMAPR-------------------------------------------------------------------    macro "MitoMAPR-1.0" {         	    if (nImages == 0) {		showMessage("No images are open.");		exit();	}	    run("Duplicate...", "title=TheOne");	selectWindow("TheOne");	run("Grays");	getDimensions(width, height, channels, slices, frames);	getPixelSize(unit, pixelWidth, pixelHeight);run("Enhance Local Contrast (CLAHE)", "blocksize=127 histogram=256 maximum=3 mask=*None*");run("Unsharp Mask...", "radius=2 mask=0.60");run("Median...", "radius=2");	run("Duplicate...", "title=Skeletor");	selectWindow("Skeletor");	run("32-bit");	run("Make Binary");	run("8-bit");getStatistics(area, Mean, min, max);	mitoArea = pow(pixelWidth, 2.0) * parseFloat(width) * parseFloat(height) * (Mean / parseFloat(max)) ;	run("Restore Selection");	getStatistics(area);	TotalCellArea=area;    MitoCoverage = ((mitoArea/TotalCellArea)*100);	run("Skeletonize");	run("Red");	selectWindow("TheOne");	run("Add Image...", "image=Skeletor x=0 y=0 opacity=100 zero");	size = toString(round(0.25*parseFloat(width)*parseFloat(pixelWidth)));	run("Scale Bar...", "width=" + size + " height=4 font=14 color=White background=Black location=[Lower Right] bold overlay");				selectWindow("Skeletor");		run("Analyze Skeleton (2D/3D)", "prune=none show display");		selectWindow("Tagged skeleton");        saveAs("Tagged skeletor.tif");        selectWindow("Skeletor-labeled-skeletons");        saveAs("labeled skeletor.tif");		close("Tagged skeleton");		close("Skeletor");		close("Skeletor-labeled-skeletons");		selectWindow("TheOne");saveAs("TheOne.tif");close("TheOne");		selectWindow("Results"); rows = nResults;		ObjectCounts = newArray(rows);		for (i=0; i<rows; i++) {			ObjectCounts[i] = getResult("# Branches", i);		}		JunctionCounts = newArray(rows);		for (i=0; i<rows; i++){			JunctionCounts[i] = getResult("# Junctions", i);		}		selectWindow("Results"); run("Close");		IJ.renameResults("Branch information", "Results");		selectWindow("Results"); rows = nResults;		ObjectLengths = newArray(rows);		for (i=0; i<rows; i++) {			ObjectLengths[i] = getResult("Branch length", i);		}		run("Close");						Objects = (parseFloat(CountForMe(ObjectCounts)))*10;		Networks = (parseFloat(countNetworksForMe(ObjectCounts)))*10;		JunctionPoints = (AddForMe(JunctionCounts))*10;		JunctionsPerNetwork = round(JunctionPoints/Networks);        MitoPartArea = mitoArea/Objects;		ObjectLength = BeMeanForMe(ObjectLengths);Maths = newArray(  "Objects(OC)",				   "ObjectLength(OL)",				   "Networks(N)",				   "JunctionPoints(JP)",			       "JunctionsPerNetwork(JP/N)",			       "MitochondrialFootprint(MF",			       "ObjectArea(OA)",			       "MitoCoverage(MC)",			       "TotalCellArea(TCA)");			     Numbers = newArray(Objects,                   ObjectLength,				   Networks,				   JunctionPoints,				   JunctionsPerNetwork,				   mitoArea,				   MitoPartArea,				   MitoCoverage,				   TotalCellArea);Units = newArray("Counts",                  unit,                 "Counts",                 "Counts",			     "Counts",			      unit+" squared",			      unit+" squared",			      "Percent",			      unit+" squared");Array.show("Data", Maths, Numbers, Units);selectWindow("Data");saveAs("Data.txt");selectWindow("Data.csv"); if (isOpen("Data.csv")) {       selectWindow("Data.csv");       run("Close");       run("Close All"); }}//Do Maths for Mefunction CountForMe(data) {	entries = data.length;	total = 0.0;	for (i=0; i<entries; i++) 		{			total = total + 1;		}		return(total);}function BeMeanForMe(data) {	entries = data.length;	total = 0.0;	for (i=0; i<entries; i++) {		total = total + data[i];	}	ave = total/parseFloat(entries);	return(ave);}function AddForMe(data) {	entries = data.length;	total = 0.0;	for (i=0; i<entries; i++) {		total = total + data[i];	}	sum = total;	return(sum);}function countNetworksForMe(data) {	entries = data.length;	total = 0.0;	for (i=0; i<entries; i++) {		if (data[i] > 1) {			total = total + 1;		}		else {		}	}	return(total);}function CountNetworkBranchesForMe(data) {	entries = data.length;	total = 0.0;	for (i=0; i<entries; i++) {		if (data[i] > 1) {			total = total + data[i];		}		else {		}	}	return(total);}B.) .IJM code for MitoMAPR-1.0_Batchvar SampleIdARRAY = newArray();var ObjectsARRAY = newArray();var JunctionsArray = newArray();var JunctionsPerNetworkArray = newArray();var NetworksARRAY = newArray();var ObjectLengthARRAY = newArray()var mitoAreaARRAY = newArray();var ObjectAreaArray = newArray();var CoverageArray = newArray();var CellAreaArray = newArray();macro "MitoMAPR Batch" {	dir = getDirectory("Choose a Directory ");	processFiles(dir);    SampleId = SampleIdARRAY;    Objects = ObjectsARRAY;    Networks = NetworksARRAY;    JunctionsPerNetwork = JunctionsPerNetworkArray;    JunctionPoints = JunctionsArray;    ObjectLength = ObjectLengthARRAY;    MitochondrialFootprint = mitoAreaARRAY;    ObjectArea= ObjectAreaArray;    MitoCoverage= CoverageArray;    TotalCellArea = CellAreaArray;	Array.show("Data",	           SampleId,	           Objects,	           Networks,	           JunctionsPerNetwork,	           JunctionPoints,	           ObjectLength,	           MitochondrialFootprint,               ObjectArea,               MitoCoverage,               TotalCellArea);}function processFiles(dir) {  list = getFileList(dir);  for (i=0; i<list.length; i++) {      if (endsWith(list[i], "/"))          processFiles(""+dir+list[i]);      else {         path = dir+list[i];         processFile(path);      }  }}function processFile(path) {    open (path);   run("Enhance Local Contrast (CLAHE)", "blocksize=127 histogram=256 maximum=3 mask=*None*");run("Unsharp Mask...", "radius=2 mask=0.60");run("Median...", "radius=2");	run("Duplicate...", "title=TheOne");	selectWindow("TheOne");		run("Grays");	getDimensions(width, height, channels, slices, frames);	getPixelSize(unit, pixelWidth, pixelHeight);   	selectWindow("TheOne");	run("Duplicate...", "title=Skeletor");	selectWindow("Skeletor");	run("32-bit");	run("Make Binary");	run("8-bit");	getStatistics(area, Mean, min, max);	mitoArea = pow(pixelWidth, 2.0) * parseFloat(width) * parseFloat(height) * (Mean / parseFloat(max)) ;	run("Restore Selection");	getStatistics(area);	TotalCellArea=area;    MitoCoverage = ((mitoArea/TotalCellArea)*100);	run("Skeletonize");	run("Red");	selectWindow("TheOne");	run("Add Image...", "image=Skeletor x=0 y=0 opacity=100 zero");	size = toString(round(0.25*parseFloat(width)*parseFloat(pixelWidth)));	run("Scale Bar...", "width=" + size + " height=4 font=14 color=White background=Black location=[Lower Right] bold overlay"); {				selectWindow("Skeletor");		run("Analyze Skeleton (2D/3D)", "prune=none show display");		close("Tagged skeleton");		close("Skeletor");		close("Skeletor-labeled-skeletons");				selectWindow("Results"); rows = nResults;		ObjectCounts = newArray(rows);		for (i=0; i<rows; i++) {			ObjectCounts[i] = getResult("# Branches", i);		}		JunctionCounts = newArray(rows);		for (i=0; i<rows; i++){			JunctionCounts[i] = getResult("# Junctions", i);		}		selectWindow("Results"); run("Close");		IJ.renameResults("Branch information", "Results");		selectWindow("Results"); rows = nResults;		ObjectLengths = newArray(rows);		for (i=0; i<rows; i++) {			ObjectLengths[i] = getResult("Branch length", i);		}		run("Close");				Objects = (parseFloat(CountForMe(ObjectCounts)))*10;		Networks = (parseFloat(countNetworksForMe(ObjectCounts)))*10;		JunctionPoints = (AddForMe(JunctionCounts))*10;		JunctionsPerNetwork = round(JunctionPoints/Networks);        ObjectArea = mitoArea/Objects;		ObjectLength = BeMeanForMe(ObjectLengths);                SampleIdARRAY = Array.concat(SampleIdARRAY, path);        ObjectsARRAY = Array.concat(ObjectsARRAY, Objects);        NetworksARRAY = Array.concat(NetworksARRAY, Networks);        JunctionsArray = Array.concat(JunctionsArray, JunctionPoints);        ObjectLengthARRAY = Array.concat(ObjectLengthARRAY, ObjectLength);        JunctionsPerNetworkArray = Array.concat(JunctionsPerNetworkArray, JunctionsPerNetwork);         mitoAreaARRAY = Array.concat(mitoAreaARRAY, mitoArea);        ObjectAreaArray = Array.concat(ObjectAreaArray, ObjectArea);        CoverageArray = Array.concat(CoverageArray,  MitoCoverage);        CellAreaArray = Array.concat(CellAreaArray, TotalCellArea);				titles = getList("image.titles");		for (i=0; i<titles.length; i++) {			selectWindow(titles[i]);			close();		}	}	 {		        SampleIdARRAY = Array.concat(SampleIdARRAY, path);        ObjectsARRAY = Array.concat(ObjectsARRAY, "");        NetworksARRAY = Array.concat(NetworksARRAY, "");        JunctionsArray = Array.concat(JunctionsArray, "");        JunctionsPerNetworkArray = Array.concat(JunctionsPerNetworkArray, "");        ObjectLengthARRAY = Array.concat(ObjectLengthARRAY, "");        mitoAreaARRAY = Array.concat(mitoAreaARRAY, "");        ObjectAreaArray = Array.concat(ObjectAreaArray, "");        CoverageArray = Array.concat(CoverageArray, "");        CellAreaArray = Array.concat(CellAreaArray, "");				titles = getList("image.titles");		for (i=0; i<titles.length; i++) {			selectWindow(titles[i]);			close();					}	}}//Do Maths for Mefunction CountForMe(data) {	entries = data.length;	total = 0.0;	for (i=0; i<entries; i++) 		{			total = total + 1;		}		return(total);}function BeMeanForMe(data) {	entries = data.length;	total = 0.0;	for (i=0; i<entries; i++) {		total = total + data[i];	}	ave = total/parseFloat(entries);	return(ave);}function AddForMe(data) {	entries = data.length;	total = 0.0;	for (i=0; i<entries; i++) {		total = total + data[i];	}	sum = total;	return(sum);}function countNetworksForMe(data) {	entries = data.length;	total = 0.0;	for (i=0; i<entries; i++) {		if (data[i] > 1) {			total = total + 1;		}		else {		}	}	return(total);}function CountNetworkBranchesForMe(data) {	entries = data.length;	total = 0.0;	for (i=0; i<entries; i++) {		if (data[i] > 1) {			total = total + data[i];		}		else {		}	}	return(total);}
C.) .IJM code for CropRdir=getDirectory("Choose a Directory");list = getFileList(dir);for (i=0; i<list.length; i++) {     if (endsWith(list[i], ".tif")){               print(i + ": " + dir+list[i]);             open(dir+list[i]);             imgName=getTitle();         ;         waitForUser ("make a selection");setBackgroundColor(0, 0, 0);run("Clear Outside");run("Crop");saveAs("Tiff",   dir +"Crop" +   imgName);close();                 run("Close All");     }}
