# Supplementary figures and images for: Neuronal TORC1 modulates longevity via AMPK and cell nonautonomous regulation of mitochondrial dynamics in C. elegans
Source: eLife. 2019 Aug 14;8:e49158. doi: 10.7554/eLife.49158 (PMC6713509; doi:10.7554/eLife.49158)

GO (Biological Process) Enrichment Analysis

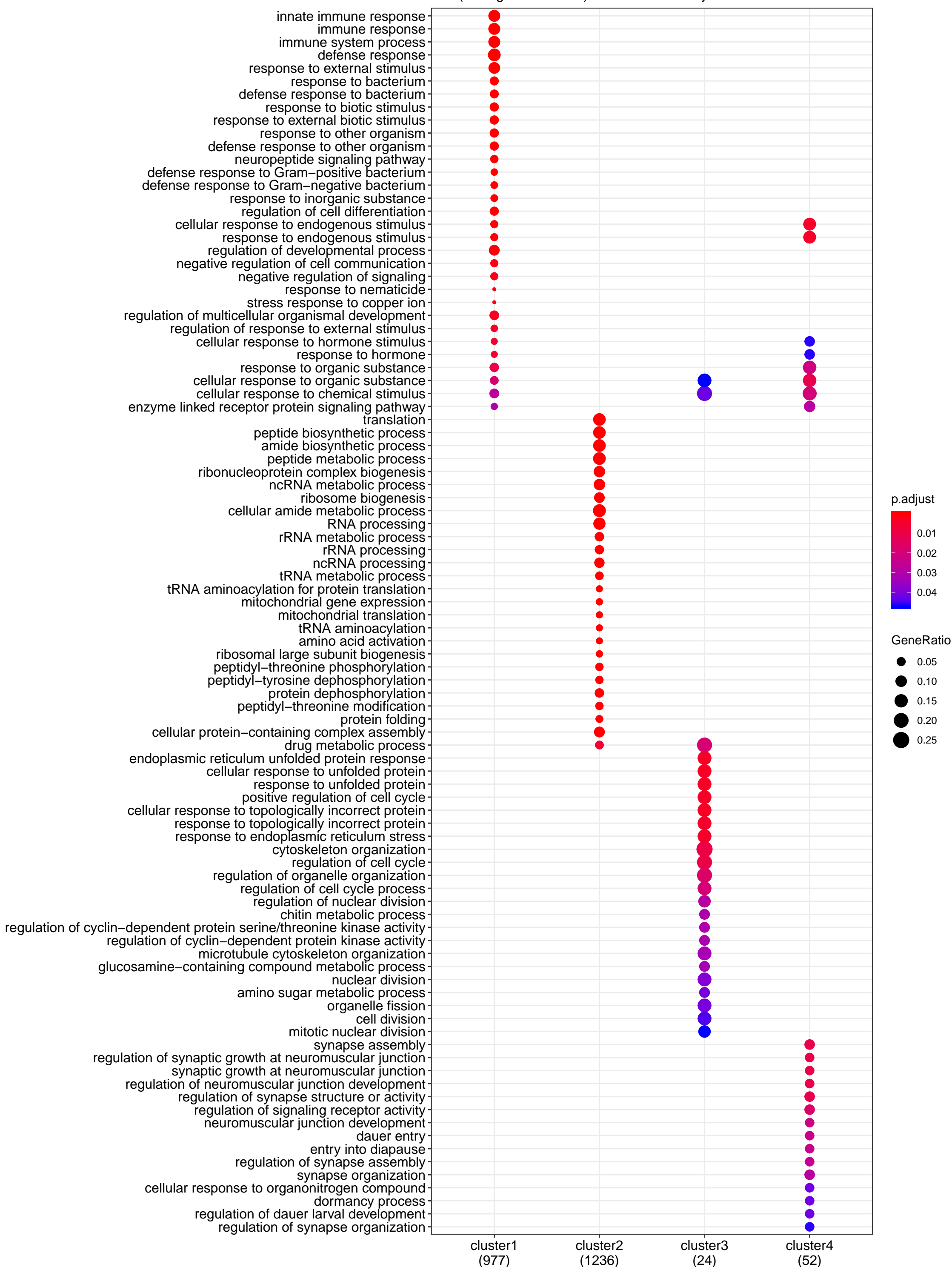

Supplement: Supplementary file 3. [file elife-49158-supp3.pdf]
