# Supplementary material for: Neuronal TORC1 modulates longevity via AMPK and cell nonautonomous regulation of mitochondrial dynamics in C. elegans
Source: eLife. 2019 Aug 14;8:e49158. doi: 10.7554/eLife.49158 (PMC6713509; doi:10.7554/eLife.49158)
Supplement: Transparent reporting form [file elife-49158-transrepform.docx]

***eLife’s* transparent reporting form**

We encourage authors to provide detailed information *within their submission* to facilitate the interpretation and replication of experiments. Authors can upload supporting documentation to indicate the use of appropriate reporting guidelines for health-related research (see [EQUATOR Network](http://www.equator-network.org/%20)), life science research (see the [BioSharing Information Resource](https://biosharing.org/)), or the [ARRIVE guidelines](http://www.plosbiology.org/article/info:doi/10.1371/journal.pbio.1000412) for reporting work involving animal research. Where applicable, authors should refer to any relevant reporting standards documents in this form.

If you have any questions, please consult our Journal Policies and/or contact us: [editorial@elifesciences.org](mailto:editorial@elifesciences.org).

**Sample-size estimation**

- You should state whether an appropriate sample size was computed when the study was being designed
- You should state the statistical method of sample size computation and any required assumptions
- If no explicit power analysis was used, you should describe how you decided what sample (replicate) size (number) to use

Please outline where this information can be found within the submission (e.g., sections or figure legends), or explain why this information doesn’t apply to your submission:

For survival analysis, we used the standard sample size of 100 animals per treatment group. In many experiments, we increased the sample size to 160-200 animals per treatment group. Sample sizes for survival assays are reported in figure legends as well as in Supplemental File 6.

For other experiments, sample sizes are listed in figure legends.

**Replicates**

- You should report how often each experiment was performed
- You should include a definition of biological versus technical replication
- The data obtained should be provided and sufficient information should be provided to indicate the number of independent biological and/or technical replicates
- If you encountered any outliers, you should describe how these were handled
- Criteria for exclusion/inclusion of data should be clearly stated
- High-throughput sequence data should be uploaded before submission, with a private link for reviewers provided (these are available from both GEO and ArrayExpress)

Please outline where this information can be found within the submission (e.g., sections or figure legends), or explain why this information doesn’t apply to your submission:

Experiments are replicated 3 times unless otherwise noted in figure legends.

Biological replicates are different animals or groups of animals. Technical replicates are protein or RNA samples from the same group of animals.

All replicates are biological replicates unless otherwise indicated in figure legends.

No animals or samples were excluded from the analyses.

Data from all lifespan experiments are included in supplemental table without excluding any lifespan replicates.

**Statistical reporting**

- Statistical analysis methods should be described and justified
- Raw data should be presented in figures whenever informative to do so (typically when N per group is less than 10)
- For each experiment, you should identify the statistical tests used, exact values of N, definitions of center, methods of multiple test correction, and dispersion and precision measures (e.g., mean, median, SD, SEM, confidence intervals; and, for the major substantive results, a measure of effect size (e.g., Pearson's r, Cohen's d)
- Report exact p-values wherever possible alongside the summary statistics and 95% confidence intervals. These should be reported for all key questions and not only when the p-value is less than 0.05.

Please outline where this information can be found within the submission (e.g., sections or figure legends), or explain why this information doesn’t apply to your submission:

For lifespan analyses, p values were calculated using Log-rank (Mantel-Cox) test.

For western blots, fluorescent reporter quantifications and qPCR, statistical significance was determined by two-tail t-test unless otherwise indicated.

P values are include in figure legends.

(For large datasets, or papers with a very large number of statistical tests, you may upload a single table file with tests, Ns, etc., with reference to sections in the manuscript.)

**Group allocation**

- Indicate how samples were allocated into experimental groups (in the case of clinical studies, please specify allocation to treatment method); if randomization was used, please also state if restricted randomization was applied
- Indicate if masking was used during group allocation, data collection and/or data analysis

Please outline where this information can be found within the submission (e.g., sections or figure legends), or explain why this information doesn’t apply to your submission:

For survival analysis, large groups of animals were prepared and ~100 animals were randomly selected per treatment group.

Masking was used in select experiments. When an experiment is replicated by an independent researcher, he/she was usually not told what treatment each group represents until data collection was finished.

**Additional data files (“source data”)**

- We encourage you to upload relevant additional data files, such as numerical data that are represented as a graph in a figure, or as a summary table
- Where provided, these should be in the most useful format, and they can be uploaded as “Source data” files linked to a main figure or table
- Include model definition files including the full list of parameters used
- Include code used for data analysis (e.g., R, MatLab)
- Avoid stating that data files are “available upon request”

Please indicate the figures or tables for which source data files have been provided:

Code for MitoMAPR analysis is included as supplemental file.

List of source data:

Figure 1b: AMPK activity is increased in raga-1 mutants

(Figure 1 — Source Data 1)

Figure 1 — figure supplement 1c: AMPK activity is increased by knockdown of TOR

(Figure 1 — Source Data 2)

Figure 1 — figure supplement 1f: The conserved S6K/Akt phosphorylation site serine 551 on AAK-2 modulates AMPK activity

(Figure 1 — Source Data 3)

Figure 2e: qPCR of raga-1 expression in SCIs and extrachromosomal lines

(Figure 2 — Source Data 1)

Figure 2 — figure supplement 3: Developmental stages of raga-1 rescue lines

(Figure 2 — Source Data 2)

Figure 3c and d: qPCR of daf-28 and ins-6

(Figure 3 — Source Data 1)

Figure 3 — figure supplement 2: qPCR validation of RNA seq results

(Figure 3 — Source Data 2)

Figure 3 — figure supplement 3: qPCR of ins-6 in day 1 adults

(Figure 3 — Source Data 3)

Figure 4c-h and Figure 4 — figure supplement 5: Effects of neuronal raga-1 rescue on parameters of muscle mitochondrial morphology

(Figure 4 — Source Data 1)

Figure 4 — figure supplement 3c-f: Effects of neuronal raga-1 rescue on parameters of neuronal mitochondrial morphology

(Figure 4 — Source Data 2)

Figure 4 — figure supplement 4c: Network states of intestinal mitochondria in raga-1 mutants

(Figure 4 — Source Data 3)

Figure 4 — figure supplement 6c-f: Mitochondria network characteristics of muscle mitochondria in unc-64 mutants

(Figure 4 — Source Data 4)
